# The development of pancreatic cancer is accompanied by significant changes in the immune response in genetically predisposed mice

**DOI:** 10.3389/fonc.2025.1603293

**Published:** 2025-06-26

**Authors:** Urszula Daniluk, Agnieszka Świdnicka-Siergiejko, Jarosław Daniluk, Małgorzata Rusak, Milena Dąbrowska, Katarzyna Guzińska-Ustymowicz, Anna Pryczynicz, Andrzej Dąbrowski

**Affiliations:** ^1^ Department of Pediatrics, Gastroenterology, Hepatology, Nutrition, Allergology and Pulmonology, Medical University of Bialystok, Bialystok, Poland; ^2^ Department of Gastroenterology and Internal Medicine, Medical University of Bialystok, Bialystok, Poland; ^3^ Department of Heamatological Diagnostics, Medical University of Bialystok, Bialystok, Poland; ^4^ Department of General Pathomorphology, Medical University of Bialystok, Bialystok, Poland

**Keywords:** pancreatic cancer, experimental mouse models, fecal microbiota transplantation, immune response, dendritic cells

## Abstract

**Purpose:**

The pathogenesis of pancreatic cancer (PC) is extremely complex and involves genetic and environmental factors, as well as significant changes in the immune response to tumor cells from the loss of immune surveillance to the development of immunotolerance to cancer. Currently available literature data on this subject is inconsistent. The purpose of our study was to evaluate the status of dendritic cells (DC) and other immune cells in the pancreas and blood of mice genetically predisposed to pancreatic cancer (Kras^G12D^ mutation). The second objective was to assess the impact of fecal microbiota transplantation (FMT) from PC mice on pancreatic tumor development and alterations in pancreatic and blood immune cell counts in mice genetically predisposed to PC.

**Methods:**

We used LSL-K-Ras^G12D^ mice, which possess the conditional knock-in mutant K-Ras^G12D^ driven by its endogenous promoter and Ela-CreERT mice, which express tamoxifen-regulated CreERT specifically in pancreatic acinar cells under the control of a full-length elastase gene promoter. The immunophenotype of immune cells separated from pancreatic tissue and circulating blood was analyzed with the use of multicolor flow cytometry and immunochemistry staining. Fecal pellets from LSL-K-Ras^G12D^ mice, that developed PC after the cerulein (CER) treatment, were collected and transplanted into animals previously treated with the antibiotic.

**Results:**

Using immunohistochemistry and flow cytometry, we found that in mice genetically predisposed to PC, cerulein (CER) administered intraperitoneally induced tumor growth and inflammatory cell infiltration in pancreatic tissue, but without affecting immune cell differentiation in the blood. In contrast, orally administered FMT activated the immune system in the gastrointestinal tract, leading to generalized immune cell activation, as observed in the blood, and local infiltration of cells in the pancreatic tissue of Kras mutant mice that developed pancreatic tumors. Interestingly, immunohistochemical evaluation of pancreatic tissue revealed that the Kras mutation alone causes increased infiltration of CD11b^+^, CD20^+^, CD3^+^, CD4^+^, and CD8^+^ cells. After FMT, there was a trend toward an increased intensity of infiltration by these immune cells, with the exception of CD11b^+^.

**Conclusions:**

Our data suggest that pancreatic cancer development in genetically predisposed mice is accompanied by profound changes in immune cell composition. Treatment with tumor-inducing agents such as CER or FMT from tumor-bearing mice, accelerated PC progression. The type of immune system response, systemic or local, in mice with pancreatic cancer depends on the route of entry of the inflammatory agent. Oral administration of FMT activated the systemic immune response, in contrast to the intraperitoneal injection of CER.

## 1 Introduction

Despite the tremendous progress that have been made in diagnosis and treatment in recent years, pancreatic cancer (PC) remains an extremely aggressive tumor with a very poor outcome and high mortality rate. Due to the lack of early clinical symptoms, local and distant organ metastases, and resistance to chemotherapy, PC is expected to be the second most common cause of cancer deaths in the United States in 2030 (1). Surgical resection is the only potentially curative treatment for PC, but because of the late manifestation of the disease, only 20% of patients are candidates for pancreatectomy and even in such cases, the 5-year survival does not exceed 25% (2, 3). In a European systemic review, the median PC survival was 4.6 months and the 5-year survival was equal to only 3% (4). One of the reasons for difficulties in early recognition of tumor and its poor treatment efficacy is our incomplete knowledge about the pathogenesis of this cancer. There is still insufficient understanding of the factors contributing to malignant transformation and expansion of a single cancer cell. In humans, there are numerous risk factors that predispose individuals to PC, including a mutation in the K-RAS gene, which is found in >90% of cases (5).

During the development of pancreatic cancer, there is a change in the character of the immune response from immune surveillance to immunotolerance. Studies on a mouse model of PC, showed that the tumor immune microenvironment (TIME) regulates the initiation and progression of cancer (6–8). Depletion of CD4^+^ T-cells leads to increased CD8^+^ T-cells infiltration and activation as well as decreased infiltration of several myeloid cell types, including macrophages and immature myeloid cells (Myeloid-derived suppressor cells, MDSCs). Consequently, it prevents pancreatic intraepithelial neoplasia (PanIN) development in genetically engineered mice which possess oncogenic K-Ras mutation in the pancreas (8). Among MDSCs, CD11b^+^ cells seemed to promote the initiation and maintenance of PC in mice with K-Ras mutation (6). Conventional dendritic cells type 2 (cDC2, CD11b^+^) were shown to present tumor-derived antigens to CD4^+^ T-cells, but then fail to support antitumor CD4^+^ T-cells differentiation (9). A strongly immunosuppressive relationship between Treg and cDC2 has been suggested, with induced Treg proliferation following cDC2 expansion and restoration of cDC2 function in the absence of Treg (9).

Recently, the influence of human microbiota on the formation of PC has been postulated, but the mechanisms of this interaction are still unknown (10, 11). Resident microbes can promote carcinogenesis by inducing inflammation, increasing cell proliferation, altering stem cell dynamics, and producing metabolites such as butyrate, which affect DNA integrity and immune regulation (12). Chronic inflammation caused by microorganisms has been shown to suppress the innate and adaptive immune response, resulting in over-activation of CD4^+^ T cells and suppression of CD8^+^ cytotoxic T cells, which ultimately promotes cancer development (11). Interestingly, in cancerous pancreatic tissue, both in mice and humans, the abundance of bacterial colonization was 1000-fold higher than in normal pancreas (11). Moreover, in animal models of PC, the composition of the gut microbiome underwent changes, as demonstrated in previous study (11, 13–15).

Taking into consideration the modulation of the immune system by microbiota, we hypothesized that microbiota may alter the dendritic cells’ (DC) function and immune response to make the host’s immunity tolerant to PC cells. The purpose of our study was to evaluate the status of DC and other immune cells in the pancreas and blood of mice genetically predisposed to pancreatic cancer (Kras^G12D^ mutation). The second objective was to assess the impact of fecal microbiota transplantation (FMT) collected from PC mice on pancreatic tumor development and changes in pancreatic and blood immune cell counts in mice genetically predisposed to PC.

## 2 Methods

### 2.1 Genetically engineered mice

In our experiment we used LSL-K-Ras^G12D^ mice, which possess the conditional knock-in mutant K-Ras^G12D^ driven by its endogenous promoter and Ela-CreERT mice, which express tamoxifen-regulated CreERT specifically in pancreatic acinar cells under the control of a full-length elastase gene promoter. LSL-Kras^G12D^ mice were bred with Ela-CreERT mice to obtain double-transgenic mice (LSL-Kras^G12D^/Ela-CreERT), from now on referred to as Kras/Cre mice, which expressed the mutant K-Ras specifically in the pancreas. We decided to use this specific transgenic animal model because, as it was previously showed, it fully replicates human PC phenotype when exposed to inflammatory stimuli (16–18). The presence of Cre recombinase does not affect the phenotype of the animals. Therefore, we used Ela-CreERT mice as controls for double transgenic Kras/Cre animals. All experiments were performed with tamoxifen-induced mice to activate Cre recombination in adult pancreatic acinar cells.

### 2.2 Treatments

Animal housing and all the experiments were performed at the Center of Experimental Medicine, Medical University of Bialystok. For experimental analyses, sex- and age-matched animals were used. All *in vivo* experiments were performed according to EU Directive 2010/63/EU and approved by the Local Committee for Experiments with the Use of Laboratory Animals, Olsztyn, Poland. Mice were subjected to a 12-h dark/light cycle at 22°C with access to water and standard rodent chow *ad libitum*.

**Figure 1:**
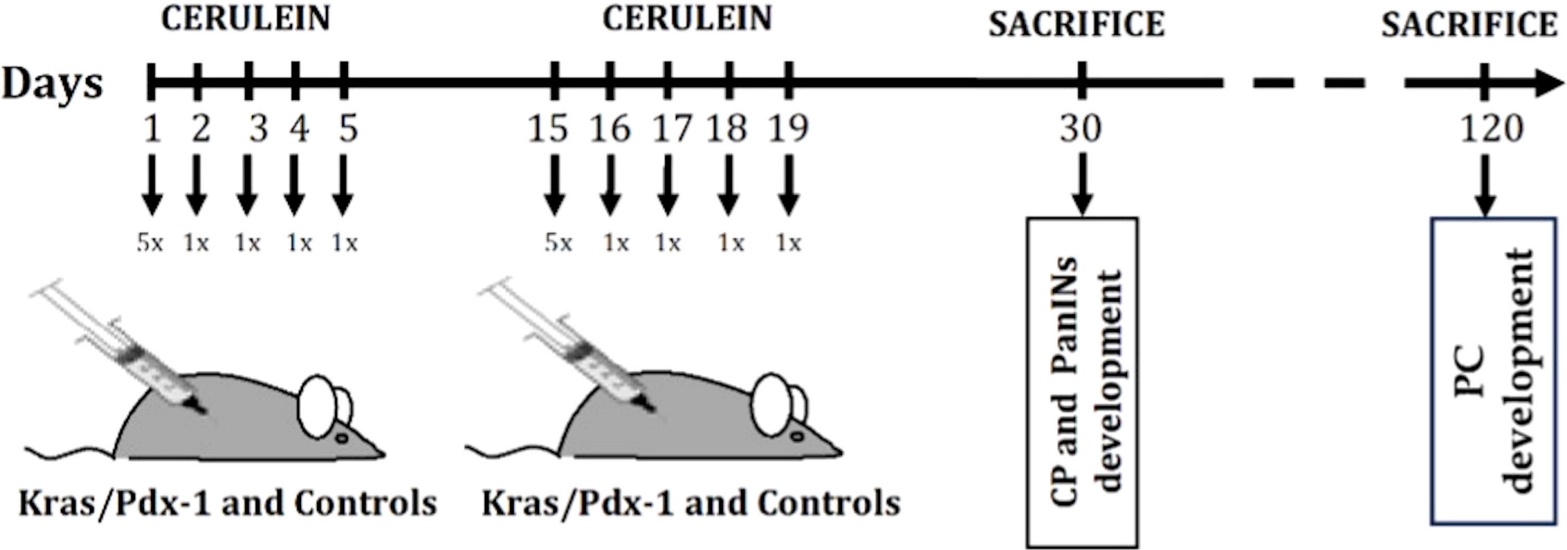
CER administration regimen for chronic pancreatitis and pancreatic cancer induction.

Based on our previous observations and literature data (16, 18), two-month-old Kras/Cre animals begin to develop pre-neoplastic lesions resembling human pancreatic intraepithelial neoplasia-1 (PanIN1). The lesions become more numerous and dysplastic (PanIN-2, PanIN-3) with age. The minority of Kras/Cre mice develop PC at one year of age. To accelerate the development of PanIN lesions and PC, animals were subjected to series of cerulein injections to induce pancreatitis, as was previously described by Daniluk et al. (16). In short, 30-day-old Kras/Cre mice (N=24) and Cre control (Cre) mice (N=24) received cerulein (CER) (50 μg/kg dissolved in 100 μl of sterile saline with 0.1% bovine serum albumin) via hourly intraperitoneal injection over 5 hours on the first day and then one injection per day for 4 consecutive days. Two weeks after the initiation of treatment, the whole course of CER injections was repeated. As controls, we used 30-day-old Kras/Cre mice (N=24) and Cre (Cre) control mice (N=24) subjected to the same saline-only injection schedule (Sal). Animals were sacrificed by a cardiac puncture at specified times points after the indicated treatments (**Figure 1**).

### 2.3 Study design

The transgenic animals and control littermates that do not express genes of interest were randomized into the following two groups (**Figure 1**): 1) development of chronic pancreatitis (CP) and pre-neoplastic (PanIN) lesions (observation period – 30 days): Kras/Cre + CER and Kras/Cre + Sal (N=12), Cre + CER or Cre + Sal (N=12); 2) Development of PC (observation period – 120 days): Kras/Cre + CER and Kras/Cre + Sal (N=12), Cre + CER and Cre + Sal (N=12). The 120-days observation period was selected based on a previous publications and our experience with PC development in transgenic mice after induction of pancreatitis with CER (16, 19).

### 2.4 Fecal microbiota transplantation

Fecal pellets from Kras/Cre mice, that developed PC after the cerulein (CER) treatment in the previous part of the study, were collected and pooled together, weighted and then placed in 0.25-1.0 ml of sterile transfer buffer. The final volume was adjusted to give 120 mg feces per ml. The fecal pellets were gently homogenized, centrifuged at 800xg for 2 min and the supernatant was collected and diluted (1:3) in sterile transfer buffer. Prior to fecal transplantation, the endogenous gut microbiota of 30 days old mice was depleted with an antibiotic solution containing streptomycin (5 mg/ml), and clindamycin (0.1 mg/ml) added to the sterile drinking water of mice for two weeks, as previously showed (10). Fecal microbiota transplantation (FMT) was performed 24 hours after antibiotics withdrawal by oral gavage of 50μl of donor fecal supernatant 3 times/week, a than once/week until the end point of the experiment. In the sham treatment group, animals received the transfer buffer only.

### 2.5 Multicolor staining for cell surface antigens and intracellular cytokines

The immunophenotype of immune cells separated from pancreatic tissue and circulating blood was analyzed with the use of multicolor flow cytometry (FACSCanto II System, BD, San Jose, USA) and immunochemistry staining. Isolation of pancreatic mononuclear cells was performed as described by Kamata et al., with minor modifications (20). Briefly, after harvesting, the pancreatic tissue was cut into ~3 mm pieces and centrifuged in PBS, followed by two washes in HBSS (1500 rpm for 5 minutes at 4°C). The tissue was then incubated in a digestion medium (437.5 mL RPMI 1640, 50 mL heat-inactivated FBS, 12.5 mL 1M HEPES) containing collagenase (20 mg/mL) and DNase I (1 mg/mL). The mixture was shaken at 150 rpm for 30 minutes at 37°C and then centrifuged (30 rcf for 5 minutes at 4°C). The resulting solution was filtered and centrifuged again (1500 rpm for 5 minutes at 4°C), and the supernatant was discarded. The pellet was resuspended in HBSS and washed by centrifugation (1500 rpm for 5 minutes at 4°C). The resulting cell pellet was used for further analysis. The blood samples were collected by cardiac puncture under general anesthesia. All experiments were performed on a cytometer BD Canto II (Beckton Dickinson). In short, freshly collected cells from blood and pancreas were labeled for 30 minutes at room temperature with fluorochrome label specific antibodies (mAbs) (all from BD Pharmingen): CD3 (145-2C11) (Catalog Number: 553064), CD4 (RM4-5) (Catalog Number: 553051), CD11b (M1/70) (Catalog Number: 557397), CD103 (M290) (Catalog Number: 557494), CD14 (rmC5-3) (Catalog Number: 553740), CD16/32 (2.4G2) (Catalog Number: 553144), CD45 (30-F11) (Catalog Number: 559864), CD25 (3C7) (Catalog Number: 553075), CD127 (SB/199) (Catalog Number: 560733), FoxP3 (MF23) (Catalog Number: 560408), T cells (cocktail CD3/CD4/CD8) (Catalog Number: 558431), B cells (cocktail CD45R/B220 (RA3-6B2)/23(FcRϵII) (B3B4)/sIgM (II/4)) (Catalog Number: 558332), and phenotyping kit Th1/Th2/Th17 (Catalog Number: 560758), IL-2 (JES6-5H4) (Catalog Number562040), IL-10 (JES5-16ES) (Catalog Number 561060), TNFalfa (MP6-XT22) (Catalog Number 561041). For intracellular cytokine assays, samples were permeabilized with BD Biosciences Perm Wash (cells were fixed with 4%PFA and permeabilized with BD Biosciences Perm Wash after surface marker staining, then 45 minutes with antibody. DC, lymphocytes, and monocytes were gated first on size and granularity and then for the relevant fluorochrome (**Supplementary Figures S1** and **S2**). The data was analyzed using Canto II cytometric software (BD). The number of cells in the tissue were calculated using the time of sample collection, the percentage of gated cells, the weight of the pancreas, and the amount of fluid in which they were suspended (fixed size 2 ml). In order to calculate absolute numbers from blood collected on EDTA, a complete blood count with smear was performed using the XN-550 analyzer (Sysmex).

### 2.6 Immunohistochemistry

Pancreata were removed immediately after the mice were sacrificed, rinsed with phosphate-buffered saline, and fixed in 10% buffered formalin overnight. Paraffin blocks with mouse pancreatic tissues were cut on the microtome into 4μm thick sections on the silanized slides. They were deparaffinised in the xylens and hydrated in alcohols. For the antigen reveal, slides were placed in citric buffer (pH=6.0) and incubated in aqueous bath for 20 minutes at 98.5°C and then for 20 minutes at room temperature. Subsequently, incubation with a 3% hydrogen peroxide to block endogenous peroxidase and a 1% bovine serum to block non-specific bonds were performed. In the next stage, the slides were incubated with rabbit anti-mouse antibodies CD11b (Biorbyt, Catalog Number: orb31554, dilution 1:100), CD20 (Biorbyt, Catalog Number: orb374711, dilution 1:200), CD3 (Biorbyt, Catalog Number: orb348965, dilution 1:100), CD4 (Biorbyt, Catalog Number: orb4830, dilution 1:100) and CD8 (Biorbyt, Catalog Number: orb323288, dilution 1:50) for 30 minutes at room temperature. To visualize the protein-antibody reaction, a polymeric technique (ImmPRESS^®^ HRP Goat Anti-Rabbit IgG Polymer Detection Kit, Vector Laboratories) and chromogen 3.3’-diaminobenzidine (ImmPACT^®^ DAB Substrate, Peroxidase (HRP), Vector Laboratories) were used. The cellular nuclei were stained with haematoxylin. The evaluation of immunohistochemical staining was performed with a light microscope using magnification of 400x and 200x for haematoxylin staining. To assess the degree of cell infiltration in pancreatic tissue, the number of CD20^+^, CD3^+^, CD4^+^ and CD8^+^ cells in 5 fields of view were counted. Otherwise, for CD11b^+^ cells immunostaining was classified as negative/weak (≤ 5 positive cells in site of view) or strong (>5 positive cells in site of view). The CD11b marker is expressed by various inflammatory cells, including macrophages, NK cells, granulocytes, and Langerhans cells. Therefore, the cell population evaluated by CD11b^+^ immunohistochemistry includes a mixture of tissue DCs and macrophages. CD11b^+^ multinucleated cells and mononuclear cells with scant cytoplasm (lymphocyte-like) were not included in the analysis.

### 2.7 Statistical analysis

Data were analyzed using Statistica 13.3 software (TIBCO Software Inc., Palo Alto, CA, USA). Statistical significance was determined by U Mann-Whitney test; p < 0.05 was considered statistically significant.

## 3 Results

### 3.1 Simultaneous interactions between oncogenic K-Ras and inflammatory factors promote development of chronic pancreatitis, precancerous lesions (PanIN 1-3), and pancreatic cancer

**Figure 2:**
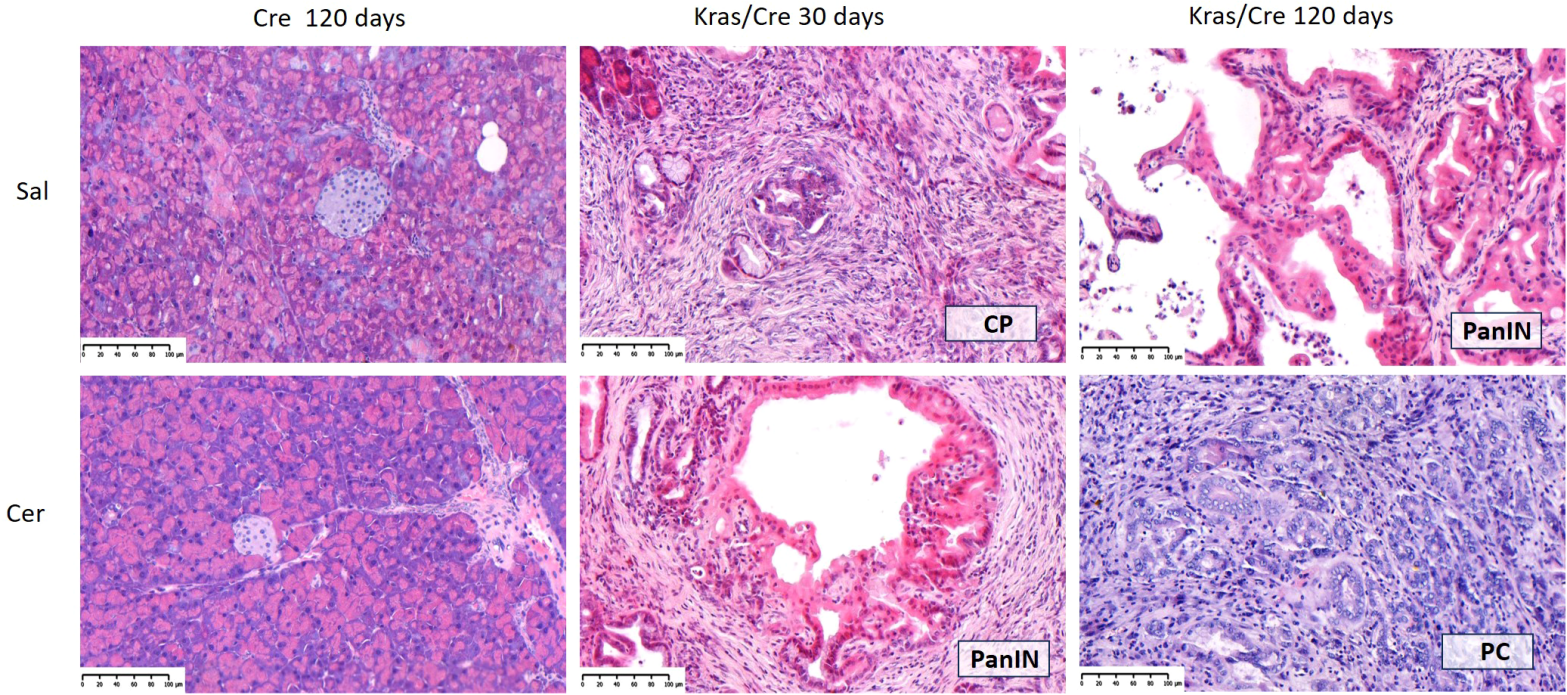
Representative hematoxylin-eosin (H&E) images of pancreas in Cre and Kras/Cre mice and percentage of pancreatic lesions among groups, n = 11–12 mice/group; scale bars represent 100 µm. Kras/Cre, mice with Kras mutation; Cre, mice without Kras mutation; Sal, saline; CER, cerulein.

To accelerate the process of inflammation and carcinogenesis in the pancreas, part of the animals were treated with CER, a CCK analog often used to generate an inflammatory process in mice resembling acute pancreatitis in humans (21). The animals were sacrificed 30 and 120 days after CER administration. In the control mice, CER administration caused only minor and transient infiltration with inflammatory cells in 25% of the animals, but this did not lead to the development of a chronic inflammatory process, precancerous or neoplastic lesions both after 30 days (data not shown) and 120 days (**Table 1**). In contrast, Kras/Cre mice 30 days after the start of the experiment showed features of CP in 55% of mice untreated with CER (**Table 1**). We found loss of pancreatic parenchyma, periacinar and perivascular fibrosis, and inflammatory cells infiltration (**Figure 2**). Moreover, CER treatment accelerated the development of neoplastic lesions. After 30 days, half of the animals developed PanIN and one developed PC among the CER-treated Kras/Cre mice. In comparison, in Sal-treated Kras/Cre mice, only one mouse developed PanIN and the development of PC was not noted (**Table 1**, **Figure 2**). After 120 days of CER administration, 67% of mice with the K-Ras mutation developed pancreatic tumor (pancreatic ductal adenocarcinoma and/or undifferentiated/anaplastic carcinoma). In contrast, only 33% of mice with the K-Ras mutation but without CER treatment showed tumor growth. Taken together, this data suggests that concomitant interaction of internal (K-Ras mutation) and external factors (inflammatory stimuli) accelerates the progression of chronic pancreatitis and ultimately the development of the cancer.

**Table 1:** Development of chronic pancreatitis (CP), preneoplastic lesions (PanIN) and pancreatic cancer (PC) in mice with and without Kras mutation.

Group	Norm (%)	CP (%)	PanIN (%)	PC (%)
1 - Cre + Sal 120 days	100.0	0.0	0.0	0.0
2 - Cre + CER 120 days	100.0	0.0	0.0	0.0
3 - Kras/Cre + Sal 30 days	36.0	55.0	9.0	0.0
4 - Kras/Cre + CER 30 days	8.0	34.0	50.0	8.0
5 - Kras/Cre + Sal 120 days	34.0	0.0	33.0	33.0
6 - Kras/Cre + CER 120 days	8.0	0.0	25.0	67.0

Kras/Cre, mice with Kras mutation; Cre, mice without Kras mutation; Sal, saline; Cer, cerulein; CP, chronic pancreatitis; PC, pancreatic cancer; PanIN, pancreatic intraepithelial neoplasia.

### 3.2 Pancreatic cancer development was accompanied by the infiltration of inflammatory cells in the pancreas but without affecting the immune cell differentiation in the blood

**Figure 3:**
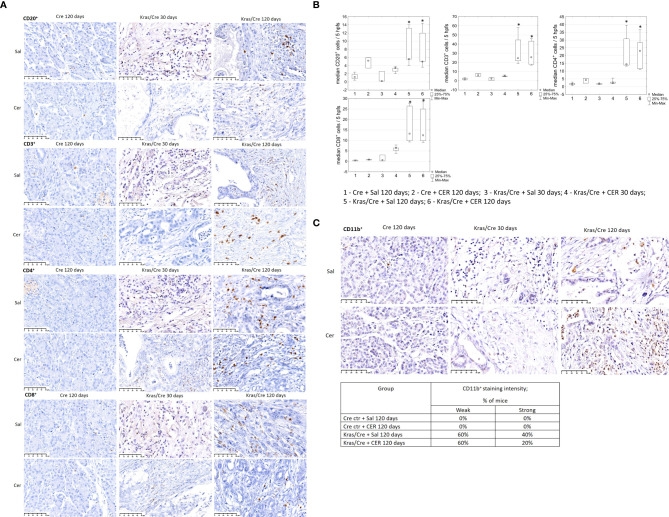
Immune cells infiltration of pancreatic tissue with PC (Kras/Cre mice) and control (Cre mice) after Sal or CER injections, **(A)** representative images of CD20^+^, CD3^+^, CD4^+^, CD8^+^ cells staining; scale bars represent 50 µm, **(B)** median number of CD20^+^, CD3^+^, CD4^+^, and CD8^+^ cells, **(C)** representative images of staining CD11b^+^ cells (scale bars represent 50 µm) and percentage of mice with CD11b^+^ cells staining, scale bars represent 50 µm, n = 5 mice/group. Kras/Cre, mice with Kras mutation; Cre, mice without Kras mutation; Sal, saline; CER, cerulein; hpfs, high power fields. Statistically significant differences (p value < 0.05) between mice are marked * (for details see the text).

Immune cell staining was performed to evaluate inflammatory cells infiltrating the pancreatic parenchyma (**Figure 3**). Immunohistochemistry analysis of pancreatic tissue revealed significant differences in the number of CD20^+^ B cells, as shown on **Figures 3A, B** (group 5 vs 1 p=0.009; group 5 vs 3 p=0.03; group 6 vs 1 p=0.03), CD3^+^ T cells (group 5 vs 1 p=0.003; group 5 vs 3 p=0.03; group 6 vs 1 p=0.006; 6 vs 3 p=0.045), CD4^+^ T cells (group 5 vs 1 p=0.005; group 6 vs 1 p=0.01), and CD8^+^ T cells (group 5 vs 1 p=0.003; group 6 vs 1 p=0.004) in the pancreas of K-Ras mutant mice with developed PC or PanIN (groups 5 and 6) compared to control mice (groups 1 and 2).

Immunochemical staining did not reveal the presence CD11b^+^ cells in the pancreas of control mice, regardless of CER stimulation (**Figure 3C**). In contrast, CD11b^+^ cells (including DCs) were present in precancerous lesions and PC of Kras/Cre mice after 120 days, independent of the CER treatment. The staining intensity of CD11b+ cells was similar in both PC and PanIN pancreas, but there were no CD11b+ cells in the pancreas of Cre mice.

**Figure 4:**
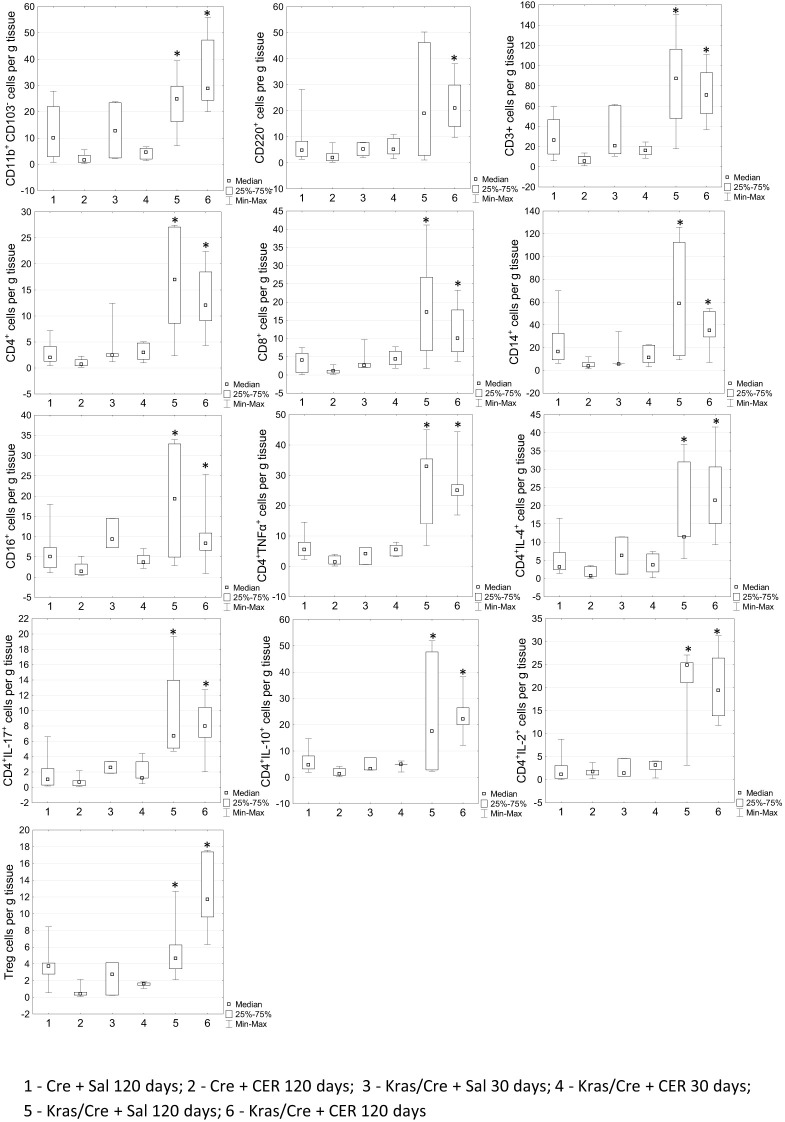
Differences in the number of chosen immune cells per gram of pancreatic tissue among the groups by flow cytometry. Kras/Cre, mice with Kras mutation; Cre, mice without Kras mutation; Sal, saline; CER, cerulein; n = 5 mice/group. Statistically significant differences (p value < 0.05) between mice are marked * (for details see the text).

To further determine the changes in number of immune cells during tumor development, we analyzed immune infiltrates in pancreatic tissue of mice with the K-Ras mutation and controls by flow cytometry (**Figure 4**). Significant differences in the number of CD11b^+^CD103^-^ DCs (group 5 vs 2 p=0.0003; group 6 vs 2 p=0.00001; group 6 vs 4 p=0.015), CD45R/B220^+^CD23^+^sIgM^+^ B cells (group 6 vs 2 p=0.00003), CD3^+^ T cells (5 vs 2 p=0.0001; 6 vs 2 p=0.000006);, CD4^+^ T cells (6 vs 1 p=0.03; 6 vs 2 p=0.00002; 5 vs 2 p=0.0001), and CD8^+^ T cells (6 vs 1 p=0.04; 6 vs 2 p=0.0001; 5 vs 2 p=0.001), T cells producing cytokines (TNFα 6 vs 1 p=0.04; 6 vs 2 p=0.000004; 5 vs 2 p=0.0006, IL-2–5 vs 1 p=0.02; 5 vs 2 p=0.04; 6 vs 1 p=0.001; 6 vs 2 p=0.005, IL-4–5 vs 2 p=0.004; 6 vs 2 p=0.00003, IL-10–5 vs 2 p=0.03; 6 vs 2 p=0.00002, IL-17–5 vs 1 p=0.03; 5 vs 2 p=0.003; 6 vs 1 p=0.01; 6 vs 2 p=0.0004), CD16^+^ NK cells (5 vs 2 p=0.003; 6 vs 2 p=0.007; 3 vs 2 p=0.04), CD14^+^ macrophages (5 vs 2 p=0.0007; 6 vs 2 p=0.0002; 1 vs 2 p=0.01) and CD4^+^CD25^+^Foxp3^+^ regulatory T cells (Treg) (5 vs 2 p=0.0157; 6 vs 2 p=0.000004; 6 vs 4 p=0.011; 1 vs 2 p=0.096) were found in the pancreas of K-Ras mutant mice with developed pancreatic tumor (group 6) or precancerous lesions (group 5) compared to control mice (groups 1 and 2) (**Figure 4**). This data indicates significant changes in the tumor’s immune microenvironment during the progression of precancerous lesions to PC.

**Figure 5:**
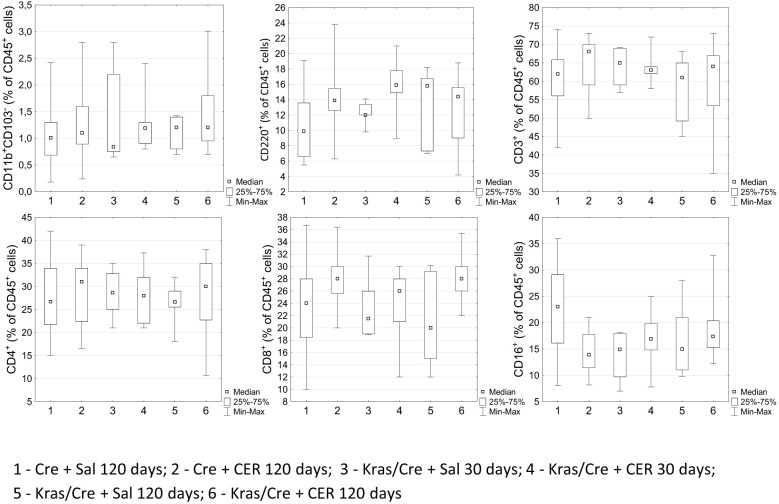
Differences in the percentages of chosen immune cells in the blood among the groups by flow cytometry. Kras/Cre, mice with Kras mutation; Cre, mice without Kras mutation; CER, cerulein; n = 8–12 mice/group.

Surprisingly, no changes in the number and profile of immune cells were observed in the blood during the process of pancreatic tumor development. The number of CD11b^+^CD103^-^ cells did not differ between mice with the K-Ras mutation and controls, regardless of the age of the animals, CER treatment and the development of PanIN or cancer (**Figure 5**). Similarly, the number of CD45R/B220^+^CD23^+^sIgM^+^ B cells, CD3^+^ T cells, CD4^+^ T cells, CD8^+^ T cells and NK cells were at the same level in Kras/Cre and Cre mice.

### 3.3 FMT-induced pancreatic tumor development was accompanied by the infiltration of inflammatory cells in the pancreas and immune cell differentiation in the blood

In a previous study, we demonstrated differences in the composition of the stool microbiota of mice with PC compared to controls and we showed that transplanting this stool into mice with the K-Ras mutation accelerated the development of PC (**Table 2**) (15).

**Table 2:** PanIN and PC development in mice with K-Ras mutation.

Group	PanIN-3	PC
Cre + sham	0%	0%
Cre + FMT	0%	0%
Kras/Cre + sham	40%	40%
Kras/Cre + FMT	37.5%	62.5%

To determine the potential mechanisms of this phenomenon, we evaluated whether FMT from PC to mice with the K-Ras mutation alters the number of immune cells in blood and pancreatic tissue after pancreatic tumor development.

**Figure 6:**
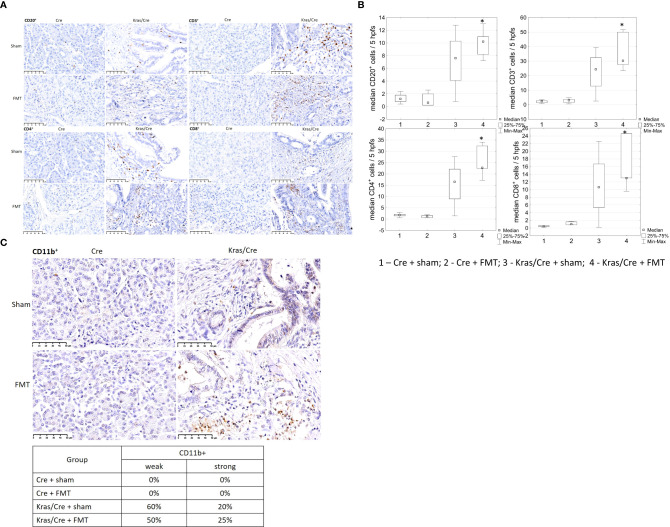
Immune cells infiltration of pancreatic tissue with PC (Kras/Cre mice) and control (Cre ctr mice) after FMT and sham treatments, **(A)** representative images of staining CD20^+^, CD3^+^, CD4^+^, CD8^+^ cells, scale bars represent 50 µm, **(B)** median number of CD20^+^, CD3^+^, CD4^+^, CD8^+^ cells; **(C)** representative images of staining CD11b^+^ cells (scale bars represent 50 µm) and percentage of mice with CD11b^+^ cells staining estimeted as a weak (≤ 5 positive cells) or strong (>5 positive cells in site of view).; Kras/Cre, mice with Kras mutation; Cre, mice without Kras mutation; FMT, fecal microbiota transplantation; n = 5 mice/group; Statistically significant differences (p value < 0.05) between mice are marked * (for details see the text).

Immunohistochemistry analysis of pancreatic tissue revealed significant differences in the number CD20^+^ B cells (group 4 vs 2 p=0.01), CD3^+^ T cells (group 4 vs 1 p=0.01, group 4 vs 2 p=0.03), CD4^+^ T cells (group 4 vs 2 p=0.003), and CD8^+^ T cells (group 4 vs 1 p=0.002) in the pancreas of K-Ras mutant mice with developed PC or PanIN (group 4) compared to control mice (groups 1 and 2) (**Figures 6A, B**). We noted no significant difference in the number of immune cells in pancreas between the group of Kras/Cre mice that developed PC or PanIN-3 after FMT (group 4) and Kras/Cre mice without FMT (group 3). A similar pattern of infiltrating CD11b^+^ cells was noted in Kras/Cre mice that developed PC or PanIN-3 after FMT, as observed in Kras/Cre mice without FMT (**Figure 6C**).

**Figure 7:**
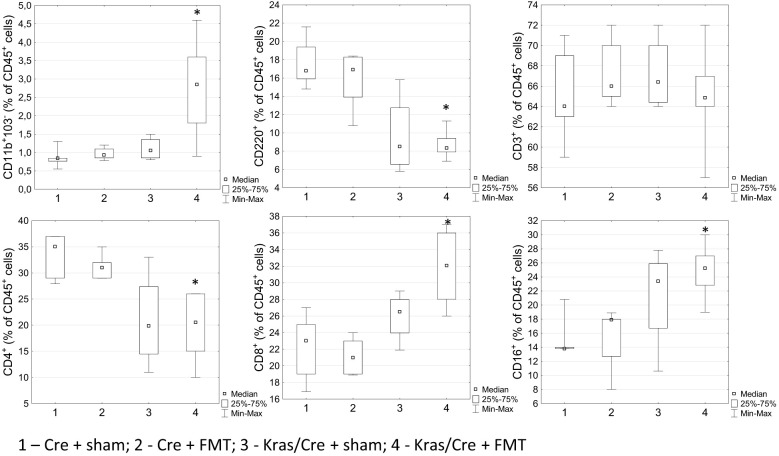
Differences in the percentage of cells in blood between groups treated or not with fecal microbiota, found by flow cytometry. Kras/Cre, mice with Kras mutation; Cre, mice without Kras mutation; FMT, fecal microbiota transplantation.; n = 6 mice/group. Statistically significant differences (p value < 0.05) between mice are marked * (for details see text).

Interestingly, analysis of immune cell profile in blood showed significant increase of CD11b^+^CD103^-^ cells in Kras/Cre mice after FMT compared to Cre mice (group 4 vs 1 p=0.01) (**Figure 7**). Another interesting result was the significant decrease of CD45R/B220^+^CD23^+^sIgM^+^ B cells (group 4 vs 1 p=0.02) and CD4^+^ T cells (group 4 vs 1 p=0.02; 4 vs 2 p=0.048) with combination of increase of CD8^+^ T cells (4 vs 2 p=0.004) and CD16^+^ NK cells (group 4 vs 1 p=0.041; group 4 vs 2 p=0.03) in Kras/Cre mice after FMT compared to Cre mice. No difference in CD3^+^ cells was found between groups. We noted no significant difference in the number of immune cells in blood between the group of Kras/Cre mice that developed PC or PanIN-3 after FMT (group 4) and Kras/Cre mice without FMT (group 3) (**Figure 7**).

## 4 Discussion

PC is accompanied by the phenomenon of immune tolerance, involving changes in the number, phenotype and function of many immune cells, including dendritic cells, which are integral part of tumor microenvironment (6–8). In our study, mice with the K-Ras gene mutation that developed pancreatic cancer, showed increased numbers of CD11b^+^CD103^-^ dendritic cells and immune cells in the pancreatic cancer tissue, but not in the blood. This phenomenon was observed independent of intraperitoneal stimulation with cerulein. However, oral administration of the gut microbiota of mice that developed PC to mice with the K-Ras mutation led to changes in the numbers of immune cells in the blood with an increase in CD11b^+^CD103^-^ DCs and CD8+ T cells with a concomitant decrease in CD4+ T cells and CD45R/B220+CD23+sIgM+ B cells during oncogenesis. We have shown that the simultaneous interaction of genetic mutations and inflammation accelerated immune cell infiltration and cancer development. This phenomenon was not observed in mice without K-Ras mutations, in which CER or FMT stimulation caused only transient inflammation that did not progress into premalignant states.

DCs are among the antigen-presenting cells (APCs) and play a key role in innate and adaptive immune processes. Due to the presence of numerous receptors on their surface, DCs, after binding to tumor antigen, become activated and present their own and foreign antigens to naive T cells, promoting antitumor T-cell response (22). The two types of conventional DCs (cDCs) are cDC1s (recognized in mice by CD103 expression), which are responsible for anti-tumor activity due to the presentation of tumor-associated antigens to CD8^+^ T cells, and cDC2s (recognized in mice by CD11b expression), an inducer of the CD4^+^ helper T cell response, and their role in the immune activity to tumor cells is not well described (23). In pancreatic cancer, the tumor immunosuppressive microenvironment (TIME) can impair DC function by reducing the number and function of DCs, producing tolerogenic and immunosuppressive cells, and preventing direct contact between DCs and tumor cells by inhibiting the production of DC-stimulating chemokines, resulting in tumor progression (23). Studies on the role of immune cells on PC development showed conflicting results, indicating how complex is the mechanism of tumor formation in the pancreas (6, 9, 24–28). The myeloid cells, including CD11b^+^ cells, were found to infiltrate pancreas with PC and deletion of these cells early during pancreatic cancer development prevents precancerous PanIN formation (6). Our study confirmed increased presence and intensity of CD11b^+^CD103^-^ cell infiltration in advanced PanIN-3 lesions and PC, which supports its crucial role in carcinogenesis. We have also shown that the presence of K-Ras mutation leads to chronic pancreatitis, a strong risk factor for PC development. Our previous study found that this may be related to prolonged activation of Ras protein in response to inflammatory stimulation (16). However, it has been suggested that chronic inflammation also promotes tumorigenesis by impairing the ability of DCs to activate the anti-tumor immune response (29). Recently reported K-Ras inhibition with the small molecule inhibitor MRTX1133 led to cancer cell clearance of advanced PC (25). MRTX1133, via Fas ligand on CD8^+^ T cells activated Fas-mediated apoptosis of cancer cells and led to a significant increase in the number of CD3^+^ T cells, CD8^+^ T cells, and CD19^+^ cells, and a decrease in CD11b^+^ myeloid infiltration (25). The presence of K-Ras mutation in our mouse model was associated with increased CD11b^+^, B and T cell abundance in the pancreas with developed tumor or precancerous lesions. The observed infiltration of immune cells was not related to tumor-inducing treatment with CER. Our results are in line with other reports of defects in the immune response to tumor cells in the pancreas (8, 24, 26). Previous studies have shown that activated CD4^+^ T or B cells can drive pathogenic inflammation and accelerate PC progression (8, 26). It has already been reported that B cells and Treg depletion has no effect on tumor progression, while CD4^+^ T cell depletion leads to a reduction in precancerous disease burden and PanIN grade (24). Interestingly, the differences in number of CD8+ and CD3^+^ cells infiltrating pancreas were noted between pancreatic ductal adenocarcinoma (PDAC) patients with short and long-term survival (10). In humans, a high density of tumor infiltrating DCs, mainly immature myeloid DCs, is indicative of a good host immune response and is a good prognostic factor in some cancers (30, 31). In contrast to mice, little or even no infiltration of cDCs in PC is found in humans, which is associated with a very long time for cancer development and its detection at a late stage, where tumor infiltration is dominated by tumor cells and necrosis (24, 32, 33). Therefore, it seems difficult to interpolate the results obtained from the animal model to humans. Our study also suggests that the presence of the K-Ras mutation does not affect the profile of immune cells in the blood. Moreover, the blood immune cell profile, including CD11b^+^CD103^-^ cells, was not associated with PC development, regardless of the administration of CER, which accelerated cancer formation. This observation unfortunately rules out the usefulness of assessing cell subsets, including DCs, in the blood as a diagnostic marker in PC in humans. Our observation is not in line with the results of a study that showed reduced blood levels of DCs in patients with PC, and that elevated blood levels of DCs were associated with better survival (29).

In our recent study, we demonstrated that FMT from mice bearing PC accelerated tumorigenesis and changed the pancreatic and stool microbial composition in mice with a Kras mutation (15). Interestingly, in the current study we showed, that oral FMT affected immune cell infiltration in pancreas with cancer compared to control mice. After FMT, there was a trend toward an increased intensity of infiltration by CD20^+^, CD3^+^, CD4^+^, and CD8^+^ cells, except for CD11b^+^. These findings are difficult to interpret based on our experiment and the currently available literature. Moreover, FMT altered the proportion of immune cells in blood of mice with PC, leading to an increase in the number of C11b^+^CD103^-^ and CD8^+^ cells and decrease in the number of CD45R/B220^+^CD23^+^sIgM^+^ and CD4^+^ T cells compared to mice without FMT intervention. This phenomenon was not observed after intraperitoneal administration of CER to Kras mutant mice that developed PC. We think that oral administration of FMT activated the systemic immune response, presumably through activation of the gut associated lymphoid tissue (GALT) leading to alteration of blood cell composition. The question of whether systemic activation of the immune response influences the course of pancreatic cancer development seems to be of great interest. The relationship between the microbiota of pancreatic cancer and the length of survival and changes in the composition of immune cells in the pancreas and blood in an animal model with orthotopic tumor was reported by Riquelme et al. (10). The results from this study suggest that gut/tumor bacteria from patients who had resected PDAC and survived long term may have a protective effect against tumors. In addition, this gut microbiome can influence the serum level of IFN-γ and IL-2 and the pancreatic tumor immune infiltrates by increasing in numbers of CD8+ T cells, as well as activated T cells (CD8+/IFNγ+ T cells). Moreover, CD8+ T cells depletion inhibited the anti-tumor effect of gut microbiome suggesting close relation between those factors (10). Recently, it has also been suggested that microbiota-derived signals can indirectly influence regulatory T lymphocytes (Tregs) by activating innate, gut-resident, antigen-presenting cells, specifically CD103^+^CD11b^+^ dendritic cells (34). Another factor that may have affected the subsets of blood cells in our study was the antibiotics used before FMT according to the study protocol. Previously, we have shown in animal model, that antibiotic therapy affects the blood profile of T and B lymphocyte subpopulations for 4 weeks (35). Many pieces of this puzzle are still missing; however, our data suggest that CD11b^+^ cells may play an important role in mediating the stimulatory effect of FMT on pancreatic carcinogenesis.

## 5 Conclusions

Our data suggest that pancreatic cancer development in genetically predisposed mice is accompanied by profound changes in immune cell composition. Treatment with tumor-inducing agents such as CER or FMT from tumor-bearing mice, accelerated PC progression. Interestingly, immunohistochemical evaluation of pancreatic tissue revealed that the Kras/Cre mutation alone causes increased infiltration of CD11b^+^, CD20^+^, CD3^+^, CD4^+^, and CD8^+^ cells. After FMT, there was a trend toward an increased intensity of infiltration by these immune cells, except for CD11b^+^. The type of immune system response, systemic or local, in mice with pancreatic cancer depends on the route of entry of the inflammatory agent. Oral administration of FMT activated the systemic immune response, presumably through GALT activation, in contrast to intraperitoneal injection of CER. The question of whether systemic activation of the immune response influences the course of pancreatic cancer development seems to be of great interest.

## Data Availability

The raw data supporting the conclusions of this article will be made available by the authors, without undue reservation.

## Supplementary material

Supplementary Figure 1 Gating strategy for flow cytometry analysis. The top panel shows a representative result of the blood analysis, and the bottom panel shows a representative result of the pancreatic tissue analysis. Dendritic cells (DCs), lymphocytes, and monocytes were initially gated based on size and granularity using forward and side scatter (green). We then identified CD45^+^ cells, and within this population, we gated for CD3^+^CD8^+^ cells (green), CD3^+^CD4^+^ cells (green), CD45R/B220^+^CD23^+^ cells (violet), and CD11b^+^CD103^−^ cells (red).

Supplementary Figure 2 Histograms for isotype controls for IL-17, IL-4, IL-10 and IL-2 staining.
