# Supplementary figures and images for: The development of pancreatic cancer is accompanied by significant changes in the immune response in genetically predisposed mice
Source: Front Oncol. 2025 Jun 26;15:1603293. doi: 10.3389/fonc.2025.1603293 (PMC12240753; doi:10.3389/fonc.2025.1603293)

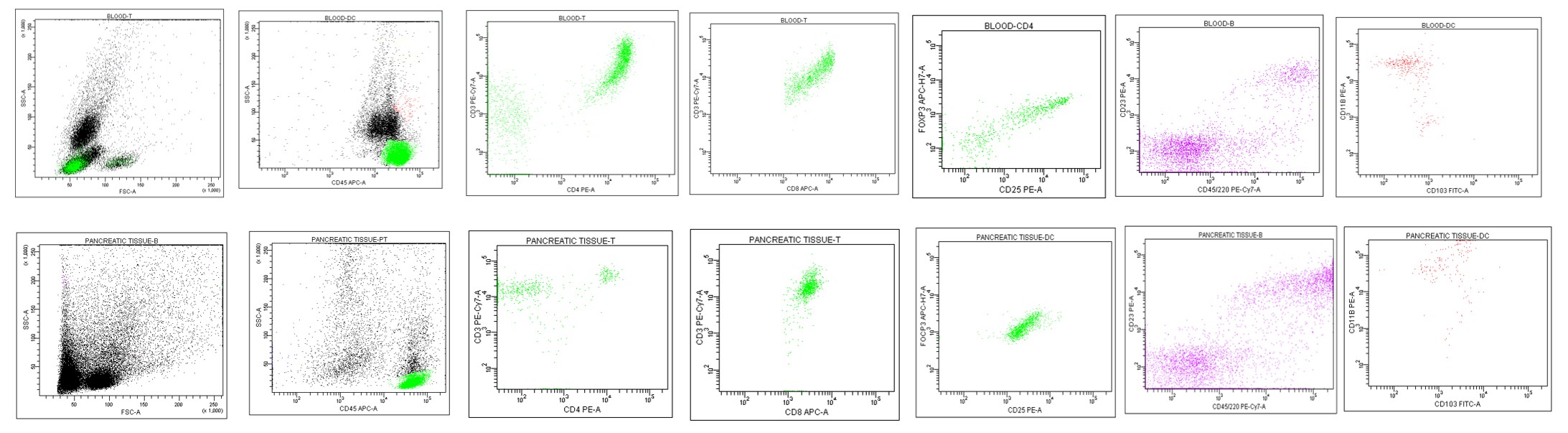

Supplement: Supplementary Figure 1 — Gating strategy for flow cytometry analysis. The top panel shows a representative result of the blood analysis, and the bottom panel shows a representative result of the pancreatic tissue analysis. Dendritic cells (DCs), lymphocytes, and monocytes were initially gated based on size and granularity using forward and side scatter (green). We then identified CD45+ cells, and within this population, we gated for CD3+CD8+ cells (green), CD3+CD4+ cells (green), CD45R/B220+CD23+ cells (violet), and CD11b+CD103− cells (red). [file Image1.jpeg]

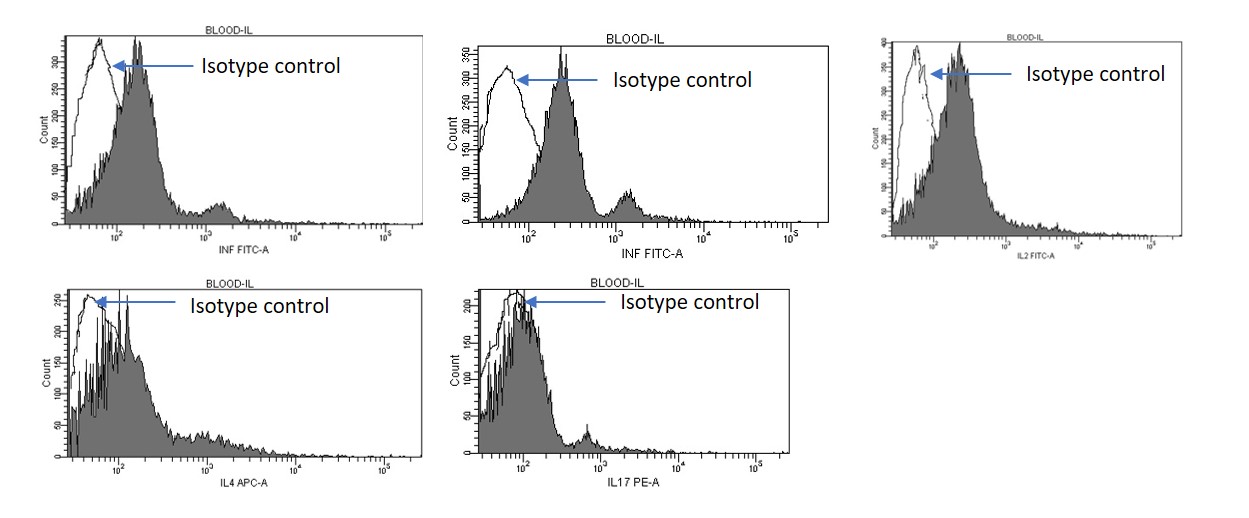

Supplement: Supplementary Figure 2 — Histograms for isotype controls for IL-17, IL-4, IL-10 and IL-2 staining. [file Image2.jpeg]
